# Antiviral Screening of Multiple Compounds against Ebola Virus

**DOI:** 10.3390/v8110277

**Published:** 2016-10-27

**Authors:** Stuart D. Dowall, Kevin Bewley, Robert J. Watson, Seshadri S. Vasan, Chandradhish Ghosh, Mohini M. Konai, Gro Gausdal, James B. Lorens, Jason Long, Wendy Barclay, Isabel Garcia-Dorival, Julian Hiscox, Andrew Bosworth, Irene Taylor, Linda Easterbrook, James Pitman, Sian Summers, Jenny Chan-Pensley, Simon Funnell, Julia Vipond, Sue Charlton, Jayanta Haldar, Roger Hewson, Miles W. Carroll

**Affiliations:** 1 Public Health England, Porton Down, Salisbury, Wiltshire SP4 0JG, UK; kevin.bewley@phe.gov.uk (K.B.); robert.watson@phe.gov.uk (R.J.W.); seshadri.vasan@phe.gov.uk (S.S.V.); andrew.bosworth@phe.gov.uk (A.B.); irene.taylor@phe.gov.uk (I.T.); linda.easterbrook@phe.gov.uk (L.E.); james.pitman@phe.gov.uk (J.P.); sian.summers@phe.gov.uk (S.S.); jenny.chan-pensley@phe.gov.uk (J.C.-P.); simon.funnell@phe.gov.uk (S.F.); julia.vipond@phe.gov.uk (J.V.); sue.charlton@phe.gov.uk (S.C.); roger.hewson@phe.gov.uk (R.H.); miles.carroll@phe.gov.uk (M.W.C.); 2 Department of Preventive and Social Medicine, Jawaharlal Institute of Postgraduate Medical Education & Research, Puducherry 605006, India; 3 Chemical Biology and Medicinal Chemistry Laboratory, New Chemistry Unit, Jawaharlal Nehru Centre for Advanced Scientific Research, Jakkur, Bengaluru 560064, Karnataka, India; chandradhish@jncasr.ac.in (C.G.); mohinimk@jncasr.ac.in (M.M.K.); jayanta@jncasr.ac.in (J.H.); 4 BerGenBio, Jonas Lies vei 91, Bergen 5009, Norway; gro.gausdal@bergenbio.com (G.G.); jim.lorens@bergenbio.com (J.B.L.); 5 Imperial College London, St Mary’s Campus, London W2 1PG, UK; jason.long08@imperial.ac.uk (J.L.); w.barclay@imperial.ac.uk (W.B.); 6 Institute of Infection and Global Health, University of Liverpool, Liverpool L69 7BE, UK; g.garcia-dorival@liverpool.ac.uk (I.G.-D.); julian.hiscox@liverpool.ac.uk (J.H.); 7 NIHR Health Protection Research Unit in Emerging and Zoonotic Infections, UK

**Keywords:** Ebola virus, antiviral, downselection, drug repurposing

## Abstract

In light of the recent outbreak of Ebola virus (EBOV) disease in West Africa, there have been renewed efforts to search for effective antiviral countermeasures. A range of compounds currently available with broad antimicrobial activity have been tested for activity against EBOV. Using live EBOV, eighteen candidate compounds were screened for antiviral activity in vitro. The compounds were selected on a rational basis because their mechanisms of action suggested that they had the potential to disrupt EBOV entry, replication or exit from cells or because they had displayed some antiviral activity against EBOV in previous tests. Nine compounds caused no reduction in viral replication despite cells remaining healthy, so they were excluded from further analysis (zidovudine; didanosine; stavudine; abacavir sulphate; entecavir; JB1a; Aimspro; celgosivir; and castanospermine). A second screen of the remaining compounds and the feasibility of appropriateness for in vivo testing removed six further compounds (ouabain; omeprazole; esomeprazole; Gleevec; D-LANA-14; and Tasigna). The three most promising compounds (17-DMAG; BGB324; and NCK-8) were further screened for in vivo activity in the guinea pig model of EBOV disease. Two of the compounds, BGB324 and NCK-8, showed some effect against lethal infection in vivo at the concentrations tested, which warrants further investigation. Further, these data add to the body of knowledge on the antiviral activities of multiple compounds against EBOV and indicate that the scientific community should invest more effort into the development of novel and specific antiviral compounds to treat Ebola virus disease.

## 1. Introduction

*Ebolavirus* is a genus of the family *Filoviridae* and includes five species: Bundibugyo virus (BDBV), Reston virus (RESTV), Sudan virus (SUDV), Taï Forest virus (TAFV) and Ebola virus (EBOV). Ebola virus is the prototype species [1,2] (formally designated Zaire ebolavirus) and was responsible for the large outbreak of Ebola virus disease (EVD) in parts of West Africa first recognized in December 2013 [3]. EBOV is the most virulent species of the family with a case mortality of up to 90%, whereas the Reston species is virtually non-pathogenic in humans [4]. In response to the outbreak in West Africa and the threat of further outbreaks in the absence of approved and proven therapeutics or vaccines, there has been increased international, political, humanitarian and scientific momentum to identify treatment strategies. In this context, during the 2013/2014 EBOV outbreak, Public Health England (PHE) was approached by several academic and commercial entities requesting rapid evaluation of repurposed drugs and experimental therapies for EBOV, using its Containment Level 4 (CL4) facilities. With support from the Ebola research funding initiative from the Wellcome Trust, a project to determine the viable drug candidates for further development was developed. The eighteen candidates in this report were selected from sixty credible leads by a scientific panel; they covered a range of potentially promising mechanisms of action against EBOV. Brief details of the compounds nominated for inclusion are outlined below:

- Ouabain: Originally used for the treatment of heart diseases [5], which has been demonstrated to reduce EBOV replication by around half when testing in vitro in a study looking into the viral protein 24 (VP24) protein and the interruption of cellular interacting proteins [6]
- 17-DMAG: An inhibitor of heat shock protein 90 (HSP90), which has been shown to reduce in vitro EBOV replication [7]
- BGB324: An inhibitor of Axl receptor tyrosine kinase, which appears to be involved with Ebola virus entry into host cells [8]
- JB1a: An antibody therapy, targeting beta-1 integrins, which have been proposed to facilitate the entry of filoviruses; treatment of target cells with the JB1a clone reduced infection using a vesicular stomatitis virus (VSIV) pseudotyped with EBOV glycoprotein [9]
- Omeprazole and esomeprazole magnesium: Members of the benzimidazoles that may stop viral entry via clathrin-mediated endocytosis by raising the endosomal pH. Both compounds were shown to inhibit lentivirus-based pseudotypes expressing EBOV glycoprotein [10]
- Gleevec and Tasigna (market names for imatinib mesylate and nilotinib, respectively): Specific tyrosine kinase inhibitors originally developed as anticancer compounds and proposed to inhibit phosphorylation of the VP40 matrix protein which is required for EBOV exit from cells [11]. During large-scale screens of antivirals against EBOV, other groups have identified Gleevec [12] and Tasigna [13] as potential EBOV inhibitors
- Aimspro (anti-inflammatory immuno-suppressive drug): Originally developed for the treatment of human immunodeficiency virus (HIV) by the production of hyperimmune serum in goats injected with inactivated HIV IIIB, the serum has revealed the presence of a range of components, including the cytokines interleukin (IL)-4 and IL-10, proopiomelanocortin, arginine vasopressin, β-endorphin and corticotropin-releasing factor [14]
- NCK-8 and D-LANA-14: Small molecules that mimic the properties of antimicrobial peptides, NCK-8 [15,16] and D-LANA-14 [17] have demonstrated potent activity against drug-resistant bacteria and their biofilms. The activity of this class of compounds is attributed to their membrane disrupting properties [18,19,20]. Peptide mimics [21] and several other small molecules have demonstrated activity against EBOV. Owing to the membrane-disrupting [22,23] modes of action of this class of compounds (e.g., NCK-8 and DLANA-14), they were expected to be active against EBOV
- Celgosivir and its prodrug castanospermine: Broad spectrum inhibitors of host glucosidases. Inhibitors of endoplasmic reticulum (ER) α-glucosidases have been shown to act as antivirals with several haemorrhagic fever viruses, including EBOV [24]
- Zidovudine, didanosine, stavudine, abacavir sulphate and entecavir: Compounds included in the study upon request of the Wellcome Trust

## 2. Materials and Methods 

### 2.1. In Vitro Screening

#### 2.1.1. Virus Assay

MRC-5 (human foetal lung) and VeroE6 (African Green monkey kidney) cells were sourced from the European Collection of Cell Cultures (ECACC) and seeded into 96-well plates. MRC-5 cells were chosen as a host-matched human cell line susceptible to EBOV infection [6] and VeroE6 for being widely used in EBOV studies [25,26,27]. Compounds were sourced commercially (Selleck Chemicals, Boston, MA, USA; and Dalton Pharm Services, Toronto, ON, Canada) wherever possible or directly from the supplier if they were not readily available (BGB324, BerGenBio, Bergen, Norway; JB1a, Avipero, Edinburgh, UK; Aimspro, Daval International, Eastbourne, UK). NCK-8 and D-LANA-14 were synthesized and characterized in Jawaharlal Nehru Centre for Advanced Scientific Research, India. Stock solutions of these compounds were made at double the final dilution to take into account an equal volume of virus suspension to be added. Several stocks were supplied in dimethyl sulfoxide (DMSO) solution. After dilution, the highest concentration of DMSO was ≤0.05%, except for omeprazole and esomeprazole, where the highest concentrations were 10% and 7.5%, respectively. 

Within the CL4 laboratory, media were removed from the inner wells of 96-well plates. Due to edge-effects, the outer wells were left with media added. Compounds were added at five replicates per dilution; three for virus addition and two mock-infected. For the first in vitro screen, EBOV suspension (strain ME718, recently renamed 1976/Yambuku-Ecran [28]) was added at a TCID_50_ (tissue culture infectious dose 50 percent) concentration of approximately 500/well to triplicate wells per compound dilution, with the remaining two wells having media alone added. Based on the inhibition of EBOV-induced cytopathic effect in different cell lines, the supernatants from MRC-5 and VeroE6 cells were harvested on days 3 and 6 post-infection, respectively. Cells were microscopically assessed for the condition of the monolayer. 

#### 2.1.2. Molecular Assay

Viral replication was measured and compared over a series of time points using a real-time polymerase chain reaction (PCR) approach. One hundred forty microliters of supernatant were added to 560 μL AVL buffer for RNA extraction in sealable deep 96-well plates for removal from the CL4 laboratory. Extraction of RNA was performed using the MagnaPure 96 small volume RNA kit (Roche, Burgess Hill, UK), a magnetic bead-based method of RNA separation. In brief, samples were vessel transferred into MagnaPure plates prior to loading onto the MagnaPure 96 automated extraction robot and RNA eluted in 60 μL nuclease-free water. Target amplification was performed using primers to Zaire ebolavirus glycoprotein as described in Trombley et al., [29] using the Fast Virus qRT-PCR Kit (Qiagen, Manchester, UK). Analysis was performed using the ABi 7500 (Applied Biosystems, Paisley, UK) under the following cycling conditions: 50 °C for 10 min, 95 °C for 30 s followed by 40 cycles of 95 °C for 15 s and 60 °C for 3 s; temperature cycling was set to the maximum ramp speed, and data were acquired and analyzed using the ABi 7500 on-board software version 2.0.6 (Applied Biosystems, Paisley, UK) with a threshold set to 0.05. Cycle threshold (Ct) values from the PCR assay were used to give a consistent reading of the amount of EBOV RNA levels in the samples. 

#### 2.1.3. Toxicity Assay

Six compounds of interest were assessed for toxicity, based on availability and results from the first in vitro screening and availability (i.e., omeprazole, esomeprazole, ouabain, 17-DMAG, Gleevec and BGB324). Serial dilutions were made and incubated on MRC-5 cells for three days. After incubation, cells were visually assessed for cytotoxicity, and monolayers were fixed with formaldehyde solution before staining with crystal violet for a gross visual inspection of cell attachment. 

#### 2.1.4. Repeat Compound Screening

Screening assays were repeated to assess for effects against EBOV using MRC-5 cells. The following changes to the previous method were employed: (i) compounds were tested in dilutions shown not to exert a cytotoxic effect on uninfected MRC-5 cells; (ii) a 10× higher viral inoculum was used (approximately 5000 TCID_50_/well); and (iii) samples were harvested after two days.

### 2.2. In Vivo Screening

Guinea pigs were used for efficacy studies [30] using group sizes of *n* = 6. Animal studies were performed under CL4 conditions with all procedures being undertaken according to the United Kingdom Animals (Scientific Procedures) Act 1986. Studies were approved by the PHE ethics committee and the Project Licence approved by a UK Home Office inspector. Vascular catheters were inserted prior to arrival to allow safe access to the intravenous route of delivery at CL4. Animals were challenged via the subcutaneous route with a dose of 10^3^ TCID_50_ guinea pig-adapted EBOV that had been passaged five times in vivo [31]. The challenge preparation was back titrated in VeroE6 cells to confirm the dose. At 6 h post-challenge, treatment was initiated with the compounds BGB324, NCK-8 and 17-DMAG. BGB324 was dissolved in 0.5% (w/w) hydroxypropyl methylcellulose/0.1% (w/w) Tween-80 to give a concentration that equated to 100 mg/kg in 1 mL. Doses of 1 mL were given orally twice daily. Compound NCK-8 was diluted with sterile water to give a concentration that equated to 5 mg/kg. One milliliter was delivered via the intravenous (i.v.) route twice daily. 17-DMAG was supplied commercially (Selleck, product S1142) and dissolved in 0.05% DMSO to give a concentration that equated to 30 mg/kg in 1 mL. Doses were delivered intraperitoneally (i.p.) and scheduled for every other day. A negative control group consisted of guinea pigs that were EBOV challenged, but received no treatment. Each day, animals were individually weighed and their temperatures were recorded by a subcutaneously-inserted temperature/identification (ID) chip. 

To prevent unnecessary animal suffering, humane clinical endpoints were used, which standardized when animals would be culled using a UK Home Office approved Schedule 1 method. The endpoints consisted of: (a) 10% weight loss and a moderate clinical symptom (e.g., lethargy, etc.); (b) 20% weight loss; or (c) showing signs of distress, as determined in consultation with the named Animal Care and Welfare Officer.

## 3. Results

### 3.1. Selection of Compounds

Due to the large number of compounds (about sixty) initially proposed for antiviral testing, a scoring assessment was undertaken to triage compounds in relation to the capacity available for testing at CL4. The assessment was based on Technical Readiness Level (TRL) (Table A1, adapted from United States Department of Defense, 2009 [32]), availability and previous evidence of efficacy against EBOV (Table A2). In addition, companies and institutions who had suggested the compounds were asked to score their drugs themselves and to provide justifications. With this information, together with information from the publicly-available literature at the time, a scientific panel identified eighteen compounds that were suitable for screening (Table 1). 

### 3.2. Effects of Compounds against In Vitro EBOV Replication

#### 3.2.1. Initial Screen at Recommended Concentration

The recommended use concentration for each compound was sought from the suppliers and/or information in the public domain. Using these concentrations, the levels of EBOV RNA after infection of two cell lines, MRC-5 and VeroE6, were assessed to give a readout of viral replication. In addition to viral RNA levels, the cytopathic effects (CPEs) were assessed for toxic effects (Table 2). Results identified nine compounds that had no reduction in viral replication despite cells remaining healthy, and these (zidovudine, didanosine, stavudine, abacavir sulphate, entecavir, JB1a, Aimspro, celgosivir, and castanospermine) were removed from further evaluation. While several compounds showed a reduction in viral RNA levels, these drugs (ouabain, 17-DMAG, omeprazole, esomeprazole magnesium, Gleevec, and Tasigna) also exhibited significant CPE in cell monolayers. However, three compounds gave reductions in the Ct value with cells remaining attached: BGB324, NCK-8 and D-LANA-14; albeit that the latter two compounds did affect the morphology of the cells to some extent. 

#### 3.2.2. Secondary Screening with a High Virus Inoculation

A repeat screening assay of compounds that showed anti-EBOV activity was conducted using MRC-5 cells only, as in the initial screen, similar responses were observed between MRC-5 and VeroE6 cells, with a higher concentration of virus inoculum and incubation for two days. Additionally, for the compounds that were previously tested at the recommended concentration, but had exerted a cytotoxic effect, the concentrations used were adjusted to determine the optimum concentration that caused tolerable toxicity after incubation on uninfected MRC-5 cells for two days. Results from this experiment confirmed the in vitro antiviral activity against EBOV for all compounds tested (Table 3).

### 3.3. Screening of Compounds for Effects against Disease in EBOV-Infected Guinea Pigs

Three of the compounds that had demonstrated in vitro activity against EBOV were screened for effects against EBOV disease in the guinea pig model: 17-DMAG, BGB324, and NCK-8. These compounds were chosen because they had caused the highest levels of viral RNA reduction, represented different modes of activities and were suitable for use in the current in vivo model without further modification or changes to ethical licensing. 

#### 3.3.1. Testing of BGB324 in EBOV-Infected Guinea Pigs

**Figure 1:**
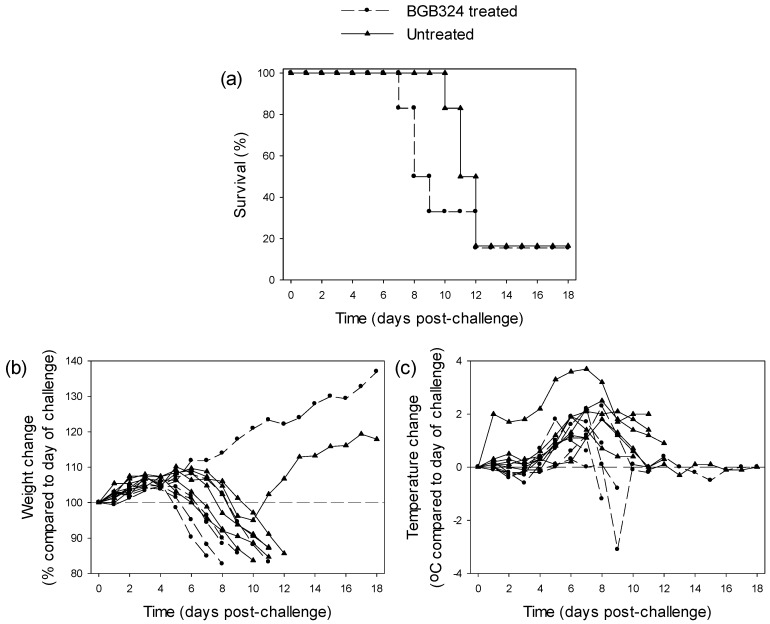
Survival and clinical parameters of guinea pigs treated with 100 mg/kg BGB324 twice daily compared to untreated controls (*n* = 6 per group). (**a**) Survival analysis after challenge with 10^3^ TCID_50_ EBOV; (**b**) Weight changes as a percentage compared to the day of challenge; (**c**) Temperature changes as °C difference compared to the day of challenge.

BGB324 was delivered orally with two doses per day starting at 6 h post-challenge. Results showed that BGB324 treatment failed to exert any statistically-significant protective effects compared to untreated animals (*n* = 6 per group; *p* = 0.358, log-rank survival analysis) (Figure 1a). Weight and temperature differences post-EBOV challenge showed that animals that met humane clinical endpoints exhibited weight loss and all animals had a rise in temperature (Figure 1b,c). However, weight loss was not observed in one animal from the BGB324-treated group, which survived until day 18 post-challenge, the scheduled end of the study. This indicates that in this animal, BGB324 exerted a protective effect.

#### 3.3.2. Testing of NCK-8 in EBOV-Infected Guinea Pigs

**Figure 2:**
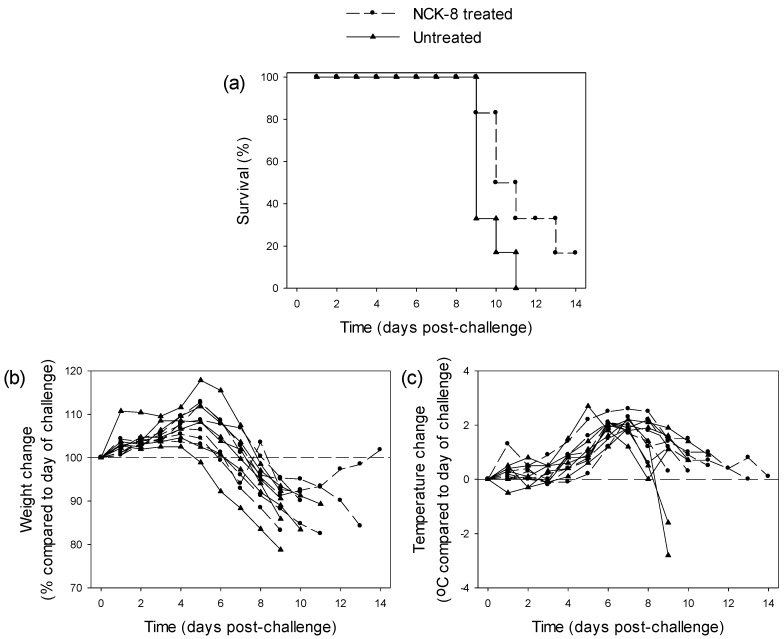
Survival and clinical parameters of guinea pigs treated with 5 mg/kg NCK-8 twice daily compared to untreated controls (*n* = 6 per group). (**a**) Survival analysis after challenge with 10^3^ TCID_50_ EBOV; (**b**) Weight changes as a percentage compared to the day of challenge; (**c**) Temperature changes as °C difference compared to the day of challenge.

NCK-8 was delivered intravenously with two doses per day starting at 6 h post-challenge. Although an increase in time to death was observed, this was not statistically significant (*n* = 6 per group; *p* = 0.076, log-rank survival analysis) (Figure 2a). All animals exhibited weight loss and had a rise in temperature (Figure 2b,c). An animal in the NCK-8-treated group began to increase its weight nine days post-challenge, indicating a recovery from EBOV infection. This animal survived to the scheduled end of the study.

#### 3.3.3. Testing of 17-DMAG in EBOV-Infected Guinea Pigs

17-DMAG was scheduled to be delivered via the intraperitoneal route every two days. However, all treated animals met humane endpoints within 24 h delivery of the first dose. Therefore, the effects of this compound against EBOV could not be ascertained in this experiment.

## 4. Discussion

The recent outbreak of EBOV disease in West Africa [3] has highlighted the urgent need for therapeutics. These needs might be met most quickly and efficiently if an existing drug with a known safety profile could be repurposed to treat EBOV effectively. Our study screened eighteen theoretically-promising antiviral therapies against EBOV using in vitro and in vivo experiments with live virus at CL4. Given the seriousness and long running extent of the West African EVD outbreak, we developed an experimental workflow to rapidly screen compounds for antiviral activity in order to focus efforts and resources on only the most promising therapies. In the first in vitro screen using 500 TCID_50_/well (multiplicity of infection (MOI) approximately 0.01), half of the compounds failed to show any activity, so these compounds were excluded from further study. For a second screen using 5000 TCID_50_/well (MOI approximately 0.1), eight of the remaining nine compounds continued to demonstrate a mean reduction in viral replication. In this screen, the drug Tasigna was not retested due to compound unavailability, but additionally, the drug Gleevec was included since it was reported to operate via the same mechanism and demonstrated better in vitro efficacy. The in vitro screens used viral RNA levels as a readout based on previous antiviral testing work [33] and infectious doses of MOI 0.01–0.1, similar to levels used in other anti-EBOV testing studies [6,34,35]. Given that the compounds tested in our study were chosen due to their perceived potential effectiveness for use against EBOV, the negative results demonstrate the importance of testing therapies using the actual live pathogen to determine effects. They also highlight the uncertainties inherent in extrapolating the mechanism of action data to drug repurposing, no matter how rational. 

Of the eight compounds that showed repeatable antiviral activity against EBOV in vitro, three were selected for in vivo studies using the guinea pig model of infection (i.e., 17-DMAG, BGB324 and NCK-8). Whereas omeprazole and esomeprazole demonstrated in vitro activity against EBOV, the results were in line with a previous report using pseudotyped viruses where the values of drug concentration causing 50% inhibition (IC_50_) were in the region of 50 μM [10]. This suggested that doses required for potent inhibition would be difficult to achieve without concomitant and significant toxicity (the licenced dosing for 40 mg esomeprazole, 20 mg esomeprazole and 20 mg omeprazole generates median maximum plasma concentrations of 1.59–9.61 μM, 0.51–4.78 μM and 0.15–3.51 μM, respectively [36]). Gleevec (brand name for imatinib mesylate) was not followed through for in vivo testing since the optimal dosing requirement (i.e., continuous dosing) was not feasible for the current set-up of the animal model at CL4. The requirement for continuous dosing is due to the short half-life in rats of a similar compound [37]. Additionally, the plasma concentrations of Gleevec usually reach 2–3 μM at normal dosages [38]. Others have suggested that the concentration for effective EBOV inhibition is 20 μM [11], which would not be possible. However, the data from the in vitro studies with live EBOV reported here show effects even when 0.74 μM was used. NCK-8 and D-LANA-14 are membrane-active small molecules that mimic the properties of natural antimicrobial peptides and may negatively impact the viral envelope and cellular lipid bilayer. Out of the two molecules, D-LANA-14, was less active; hence its efficacy was not assessed in the animal model. The guinea pig model of EBOV disease was used in this work since it presents a robust, accessible and broadly reflective rodent model for the screening of countermeasures [39]. Importantly, the model also allows catheterized animals to be used, allowing direct access to the intravenous route. This is the preferred route of delivery for many antiviral compounds and is compatible for work at the highest microbiological containment [30].

No positive effects of BGB324 against EBOV infection in guinea pigs were observed in relation to survival. Both BGB324-treated and untreated controls exhibited 83% mortality. However, the survivor in the BGB324-treated group did not show the significant loss in weight that is typical of EBOV-infected guinea pigs [31] and that was exhibited by the survivor in the untreated group. Both surviving guinea pigs exhibited an increase in temperature typical of EBOV infection in this model [31]. It is possible that these observations are a consequence of broad guinea pig responses, since these animals were outbred individuals and there were subtle differences between their responses. The effects of BGB324 were proposed to be due to the inhibition of tyrosine kinase inhibitors [40]. Thus, it could be speculated that other tyrosine kinase inhibitors, including the two in this study (Gleevec and Tasigna), would also not have demonstrated protective effects.

Treatment of EBOV-challenged guinea pigs with NCK-8 was the only instance that showed an effect on disease progression, with a demonstrable increased mean time to death; albeit, this was not statistically significant. All animals treated with NCK-8 exhibited weight loss and temperature increases, in line with the levels of untreated animals. Interestingly, this class of compounds has been tested against bacterial pathogens [15,19,41] and has been found to be quite effective. Other peptide mimics have demonstrated antiviral activity against EBOV, as well [21]. However, it should be noted that although NCK-8 showed high levels of antiviral activity, the cell monolayers were phenotypically different for both VeroE6 and MRC-5 cell lines. This observation warrants further investigation, especially the dependence of activity on the concentration of the compounds. Nevertheless, NCK-8 was well-tolerated when tested in guinea pigs and led to the recovery of one animal from EBOV infection.

When tested in guinea pigs, the effects of 17-DMAG could not be clearly ascertained since these animals met humane endpoints prior to disease symptoms of EBOV disease. The dose used in our study was 30 mg/kg; whereas there are no reports of 17-DMAG being used in guinea pigs, others have shown that 75 mg/kg delivered orally was well tolerated in mice and rats [42]. Therefore, it is likely that there is a non-compatible interaction with a component of guinea pig physiology that results in a toxic effect. To further test the effectiveness of 17-DMAG against EBOV, an animal system where the compound has shown to be non-toxic should be used, such as the mouse. However, due to the mouse model not being as relevant to human disease as the guinea pig [43], the value of conducting such studies may be counterproductive.

In summary, our results provide details on the antiviral properties of eighteen potential therapies. Whereas the experiments were limited and could have been extended to include viable viral loads by plaque assay or modern methods for the assessment of cytotoxicity, the studies were conducted to provide rapid results during an active EBOV outbreak and within the limitations of CL4 facilities where all handling of live EBOV was within a specialized cabinet-line system. Candidates for this study were chosen via a project-specific selection committee (comprising of members from PHE, academia and the Wellcome Trust), not by screening approaches of a large number of compounds [12,44,45], so were more selective based on perceived activity against EBOV. However, the value of this work is the use of live EBOV, providing valuable insight into compounds that warrant further investigation and, as important, those that showed no antiviral effects against EBOV.

## 5. Conclusions 

In conclusion, the data in this report will help to inform decisions on which compounds should be investigated further and, equally importantly, which ones should not. Given the limited facilities and restrictions of working with live Hazard Group 4 viruses, the focus should now be on treatments that have shown promise with live EBOV using in vitro and in vivo models. 

## Figures and Tables

**Table 1:** Scoring of selected compounds for Ebola virus (EBOV) screening. TRL, Technical Readiness Level.

Name	TRL Score ^1^	Availability ^2^	Efficacy ^3^	Total
Ouabain	4	2	1	7
17-DMAG	4	2	1	7
BGB324	4	2	1	7
Zidovudine	4	2	1	7
Didanosine	4	2	1	7
Stavudine	4	2	1	7
Abacavir sulphate	4	2	1	7
Entecavir	4	2	1	7
JB1a	3	2	1	6
Omeprazole	3	2	1	6
Esomeprazole magnesium	3	2	1	6
Gleevec	3	2	0.5	5.5
Aimspro	3	2	0	5
NCK-8	3	2	0	5
D-LANA-14	3	2	0	5
Tasigna	3	1	0.5	4.5
Celgosivir	2	2	0	4
Castanospermine	2	2	0	4

^1^ Scored from 1 to 9 (TLR table; Appendix A). ^2^ Availability for use in the clinic. ^3^ Previous data on efficacy against EBOV.

**Table 2:** Changes in EBOV RNA levels and cell health in MRC-5 and VeroE6 cells treated after infection with compounds at the recommended concentrations.

Name	Concentration	MRC-5		VeroE6	
		Ct Difference ^1^	Cell Appearance ^2^	Ct Difference	Cell Appearance
Ouabain	20 nM	3.48 ± 0.21	x	−3.73 ± 4.88	x
17-DMAG	5 μM	3.72 ± 0.18	x	−0.63 ± 1.39	x
BGB324	3 μM	3.05 ± 0.75	✓	−1.83 ± 1.13	✓
Zidovudine	5 μM	−3.12 ± 0.27	✓	−7.91 ± 2.67	✓
Didanosine	5 μM	−0.43 ± 3.87	✓	−2.52 ± 1.27	✓
Stavudine	5 μM	−2.87 ± 0.22	✓	−3.93 ± 0.25	✓
Abacavir sulphate	5 μM	−1.54 ± 3.26	✓	−3.95 ± 2.69	✓
Entecavir	5 μM	−3.08 ± 0.20	✓	−4.44 ± 1.11	✓
JB1a	2 μg/mL	−4.02 ± 0.13	✓	−5.48 ± 0.50	✓
Omeprazole	100 μM	1.35 ± 1.35	x	2.21 ± 1.08	x
Esomeprazole magnesium	75 μM	1.05 ± 0.79	x	1.62 ± 0.36	x
Gleevec	20 μM	3.60 ± 0.63	x	3.49 ± 0.54	x
Aimspro	Neat	−2.03 ± 0.95	✓	−4.60 ± 1.15	✓
NCK-8	1 mg/mL	>10	*	>10	*
D-LANA-14	1 mg/mL	>10	*	>10	*
Tasigna	20 μM	3.59 ± 0.57	x	−0.13 ± 0.33	✓
Celgosivir	200 μM	−2.52 ± 0.21	✓	−2.41 ± 0.12	✓
Castanospermine	200 μM	−1.58 ± 3.23	✓	−0.26 ± 4.11	✓

^1^ Difference between mean value of untreated cells (*n* = 3) versus treated cells (*n* = 3). A positive value indicates a reduction in viral RNA levels. Values shown are mean of triplicates ± standard deviation. ^2^ Presence of healthy and adherent cells. Asterisks indicate that cells were attached, but with a changed morphological appearance.

**Table 3:** Changes in EBOV RNA levels in MRC-5 cells treated with compounds at three dilutions determined not to cause cytotoxicity in non-infected treated cells.

Name	Concentration	Ct Difference ^1^
Ouabain	20 nM	0.06 ± 0.10
	6.7 nM	0.09 ± 0.23
	2.2 nM	0.33 ± 0.35
17-DMAG	63.3 nM	0.30 ± 0.20
	21.1 nM	0.26 ± 0.57
	7.0 nM	0.44 ± 0.06
BGB324	1 μM	0.90 ± 0.15
	0.3 μM	0.67 ± 0.09
	0.1 μM	0.34 ± 0.05
Omeprazole	100 μM	0.70 ± 0.10
	33.3 μM	0.77 ± 0.31
	11.1 μM	0.86 ± 0.22
Esomeprazole	25 μM	0.78 ± 0.25
	8.3 μM	0.50 ± 1.06
	0.93 μM	0.17 ± 0.16
Gleevec	6.7 μM	1.55 ± 0.20
	2.2 μM	1.03 ± 0.42
	0.74 μM	0.64 ± 0.03
NCK-8	150 μg/mL	1.54 ± 0.44
	50 μg/mL	1.33 ± 0.09
	16.7 μg/mL	1.09 ± 0.17
D-LANA-14	60 μg/mL	0.96 ± 0.19
	20 μg/mL	0.37 ± 0.11
	6.7 μg/mL	0.40 ± 0.32

^1^ Difference between the mean value of untreated cells (*n* = 3) versus treated cells (*n* = 3). A positive value indicates a reduction in viral RNA levels. Values shown are the mean of triplicates ± standard deviation.

## Appendix

**Table A1:** Technical Readiness Level (TRL) scoring.

U.S. Army Medical Research and Material Command (USAMRMC) equivalent TRL descriptions for drugs, biologics and vaccines (pharmaceuticals) synthesized from the Technology Readiness Assessment (TRA) Deskbook [46].			
**TRL**	**Description**	**Decision Criterion**	**Supporting Information**
**1**	Lowest level of technology readiness. Maintenance of scientific awareness and generation of scientific and bioengineering knowledge base. Scientific findings are reviewed and assessed as a foundation for characterizing new technologies.	Scientific literature reviews and initial market surveys are initiated and assessed. Potential scientific application to defined problems is articulated.	Reviews of open, published scientific literature concerning basic principles. Findings from market surveys of the open literature. Privately funded research findings or market surveys are proprietary and rarely available to the public but could be made available upon request under confidentiality agreement.
**2**	Intense intellectual focus on the problem, with generation of scientific “paper studies” that review and generate research ideas, hypotheses, and experimental designs for addressing the related scientific issues.	Hypothesis(es) is (are) generated. Research plans and/or protocols are developed, peer reviewed, and approved.	Focused literature reviews are conducted and scientific discussions are held to generate research plans and studies that identify potential targets of opportunity for therapeutic intervention and to facilitate strategic planning. Supporting analyses provide scientific information and data for developing research proposals for filling in data gaps and identifying candidate concepts and/or therapeutic drugs. Documented by peer-reviewed approved protocol(s) or research plan(s).
**3**	Basic research, data collection, and analysis begin in order to test hypothesis, explore alternative concepts, and identify and evaluate critical technologies and components supporting research and eventual development of the pharmaceutical candidate, identification of sites and mechanisms of action (and potential correlates of protection for vaccines), as well as initial characterization of candidates.	Initial proof-of-concept for candidate constructs is demonstrated in a limited number of in vitro and in vivo research models.	Documentation of the results of laboratory studies demonstrates preliminary proof-of-concept (with candidate constructs) from in vitro and animal studies.
**4**	Non- good laboratory practice (GLP) research to refine hypothesis and identify relevant parametric data required for technological assessment in a rigorous (worst case) experimental design. Exploratory study of candidate drugs or of critical technologies for effective integration into candidate biologic/vaccine constructs. Candidate drugs (or biologics/vaccines) are evaluated in animal model(s) to identify and assess safety, toxicity and adverse/biological/side effects, and assays (and/or surrogate markers and endpoints) to be used during non-clinical and clinical studies to evaluate and characterize candidate pharmaceuticals are identified.	Proof-of-concept and safety of candidate drug formulations (or candidate biologic/vaccine constructs) are demonstrated in defined laboratory/animal model(s).	Documented proof-of-concept and safety of the candidate are demonstrated by results of formulation studies (or proposed production/purification methods of the biologic/vaccine), laboratory tests, pharmacokinetic studies, and selection of laboratory/animal models.
**5**	Intense period of non-clinical and preclinical research studies involving parametric data collection and analysis in well-defined systems, with pilot lots of candidate pharmaceuticals produced and further development of selected candidate(s). In the case of drug, results of research with pilot lots provide basis for a manufacturing process amenable to current good manufacturing practice (cGMP)-compliant pilot lot production. In the case of biologic/vaccine, research results support proposing a potency assay, proposing a manufacturing process amenable to cGMP-compliant pilot lot production, identifying and demonstrating proof-of-concept for a surrogate efficacy marker in an animal model(s) applicable to predicting protective immunity in humans, and demonstrating preliminary safety and efficacy against an appropriate route of challenge in a relevant animal model. Conduct GLP safety and toxicity studies in animal model systems. Identify endpoints of clinical efficacy or its surrogate. Conduct studies to evaluate pharmacokinetics (PK) and pharmacodynamics (PD) and/or immunogenicity as appropriate. Stability studies initiated.	A decision point is reached at which it is determined that sufficient data on the candidate pharmaceutical exist in the draft technical data package to justify proceeding with preparation of an investigational new drug (IND) application.	Reviewers confirm adequacy of information and data in draft technical data package to support preparation of an IND application. Documentation in the draft technical data package contains data from animal pharmacology and toxicology studies, proposed manufacturing information, and clinical protocols suitable for phase 1 clinical testing.
**6**	Pre-IND meeting (type B) held and IND application prepared and submitted to the Center for Drug Evaluation and Research (CDER) or the Center for Biologics Evaluation and Research (CBER). Phase 1 clinical trials are conducted to demonstrate safety of candidate in a small number of subjects under carefully controlled and intensely monitored clinical conditions. Evaluation of PK and PD (and/or immunogenicity) data to support the design of well-controlled, scientifically valid phase 2 studies. Production technologies for drug candidates are demonstrated through production-scale cGMP plant qualification, and surrogate efficacy models for biologics/vaccines are validated.	Data from phase 1 clinical trials meet clinical safety requirements and support proceeding to phase 2 clinical trials.	For phase 1 clinical trials to begin, the following are needed: the Food and Drug Administration (FDA)’s and sponsor’s summary minutes of pre-IND meeting document agreements and general adequacy of information and data to support submission of IND application. Review of the submitted IND application does not result in a FDA decision to put a clinical hold on phase 1 clinical trials with the candidate pharmaceutical. For entry into phase 2 clinical trials, the results from phase 1 clinical studies have to demonstrate safety of candidate pharmaceutical. An updated IND, amended with a new clinical protocol to support phase 2 clinical trials or surrogate test plan and submitted to the FDA, documents achieving this criterion.
**7**	Phase 2 clinical trials are conducted to determine activity/efficacy/immunogenicity/safety/toxicity of the pharmaceutical as appropriate. These and/or PK-PD data are used to establish product final dose, dose range, schedule, and route of administration. Data are collected, presented and discussed at pre-phase 3 (or surrogate efficacy] meeting (type B) with CDER (or CBER) in support of continued drug development of the drug (or biologic/vaccine), and clinical endpoints and/or surrogate efficacy markers and test plans agreed.	Phase 3 clinical study plan or surrogate test plan has been approved.	FDA’s summary minutes of pre-phase 3 meeting with sponsor discussing results of phase 1 and phase 2 trials, as well as protocols or test plans, provide a record of agreements and basis for sponsor to proceed with phase 3 clinical study or surrogate test plan. An updated IND application, amended with a new clinical protocol to support phase 3 clinical trials or surrogate test plan and submitted to the FDA, documents achieving this criterion.
**8**	Implementation of expanded phase 3 clinical trials or surrogate tests to gather information relative to the safety and effectiveness of the candidate drug/biologic/vaccine. Trials are conducted to evaluate the overall risk–benefit of administering the candidate product and to provide an adequate basis for labelling. Process validation is completed and followed by lot consistency/reproducibility studies. In the case of a drug, New Drug Application (NDA) is submitted to CDER following pre-NDA meeting (type B). In the case of a biologic/vaccine, Biologics License Application (BLA) is prepared and submitted to CBER following pre-BLA meeting (type B). Facility Preapproval Inspection (PAI) is completed.	Approval of the NDA for drug by CDER, or approval of the BLA for biologics/vaccines by CBER.	FDA issuance of an approval letter after their review of the NDA or BLA application submitted by the sponsor for the pharmaceutical documents this criterion.
**9**	The pharmaceutical (i.e., drug/biologic/vaccine) can be distributed/marketed. Post-marketing studies (non-clinical or clinical) may be required and are designed after agreement with the FDA. Post-marketing surveillance.	None. Continue surveillance.	FDA transmits any requirement for post-marketing studies. Begin post-approval reporting requirements. Maintain cGMP compliance.

**Table A2:** Additional Scoring Criteria

Points	Description	Decision Criteria	Supporting Info
**0 or 1 or 2**	Availability to make a difference to the current epidemic	Repurposed pharmaceutical or the ability to generate and supply sufficient material within two weeks for in vitro and in vivo studies, and if required subsequently, a reasonable number of therapeutic doses to make a difference to the current Public Health Emergency of International Concern declared by the World Health Organization (WHO).	Documentation to prove that the candidate is: a pharmaceutical licensed in a well-regulated (e.g., FDA, Medicines and Healthcare products Regulatory Agency (MHRA)) market that does not require further development and is widely available (score of 2); or used or approved for use in clinical trials or patients (e.g., under Emergency Use Authorization or Expanded Access/ Compassionate Use) with guaranteed availability (score of 2); or to be supplied as outlined in the decision criterion, with reliable estimates on the feasibility, costs, timeline, etc. (score of 1). Thus, the level/quality of existing data package, any licensure/indications in humans, commitment/track record of the developers, and the feasibility to supply sufficient doses for the study and subsequently to solve the current epidemic should be well-documented.
**0 or 0.5 or 1 or 2**	Likely efficacy against the pathogenic microorganism of interest.	Prior efficacy data against the pathogenic microorganism (or a related agent) of interest, for example through reduction of load or host-immune response.	Scientific studies, reports, commercial-in-confidence information documenting evidence (e.g., theoretical or in silico, in vitro and/or in vivo data) that the candidate is likely to be efficacious against the pathogenic microorganism (or a related agent) of interest. Suggested score: in silico (0.5); in vitro (1); in vivo (2)
**0 or 1 or 2**	Practicality and cost-effectiveness (tie-breaker)	Is it a practical and cost-effective solution for frontline clinical response to the current, as well as future epidemics?	Documentation to prove that: the current or likely formulation will not be complex (e.g., oral, intravenous (i.v), multiple dosing), and any transition from the existing gold standard treatment(s) would not be too difficult; and the candidate would or could be a cost-effective solution, especially to make a difference to patients affected the current epidemic.
